# Interaction between N-cadherin and decoy receptor-2 regulates apoptosis in head and neck cancer

**DOI:** 10.18632/oncotarget.25846

**Published:** 2018-07-31

**Authors:** Phuong Thao Nguyen, Dung Nguyen, Chanbora Chea, Mutsumi Miyauchi, Makiko Fujii, Takashi Takata

**Affiliations:** ^1^ Department of Oral and Maxillofacial Pathobiology, Basic Life Science, Institute of Biomedical and Health Sciences, Hiroshima University, Hiroshima, Japan; ^2^ Department of General Internal Medicine, Hiroshima University Hospital, Hiroshima, Japan; ^3^ Department of Global Dental Medicine and Molecular Oncology, Integrated Health Sciences, Institute of Biomedical and Health Sciences, Hiroshima University, Hiroshima, Japan

**Keywords:** N-cadherin, DR-5, DcR-2, anti-apoptosis, death receptors

## Abstract

N-cadherin is a neural cell adhesion molecule that aberrantly occurs in head and neck cancers to promote cancer cell growth. However, the underlying mechanisms remain unclear. Here we report that N-cadherin increases cancer cell growth by inhibiting apoptosis. Apoptosis eliminates old, unnecessary, and unhealthy cells. However, tumor cells have the ability of avoiding apoptosis that increases cancer cell growth. Recent studies have found that tumor necrosis factor-related apoptosis-inducing ligand (TRAIL) selectively induces apoptosis in tumor cells by reacting with four distinct cell surface receptors: TRAIL-R1 (DR-4), TRAIL-R2 (DR-5), TRAIL-R3 (DcR-1), and TRAIL-R4 (DcR-2). Among these TRAIL receptors, the death receptors DR-4 and DR-5 transmit apoptotic signals owing to the death domain in the intracellular portion. Conversely, the decoy receptors DcR-1 and DcR-2 lack a complete intracellular portion, so neither can transmit apoptotic signals. DcR-1 or DcR-2 overexpression suppresses TRAIL-induced apoptosis.

In this study, N-cadherin overexpression increased DcR-2 expression and decreased DR-5 expression. In contrast, knockdown of N-cadherin expression upregulated DR-5 expression and downregulated DcR-2 expression. A significantly positive relationship between N-cadherin and DcR-2 expression was also found in HNSCC specimens. Those specimens with a lower apoptotic index showed a higher expression of N-cadherin and/or DcR-2. In addition, we demonstrated that N-cadherin interacts directly with DcR-2. Notably, DcR-2 induces cancer cell survival through the cleavage of caspases and PARP by activating MAPK/ERK pathway and suppressing NF-kB/ p65 phosphorylation, which has a very important role in resistance to chemotherapy.

## INTRODUCTION

Apoptosis is a crucial process in the development and homeostasis of normal tissue. It can be activated by intrinsic cellular pathways or various extrinsic stimuli, such as ultraviolet radiation, γ-irradiation, and chemotherapeutic drugs, and extracellular ligands, particularly members of the tumor necrosis factor (TNF) ligand family. In turn, TNF proteins stimulate death receptors (DRs), resulting in the transduction of either apoptotic or survival signals [1, 2]. Recent studies have found that tumor necrosis factor-related apoptosis-inducing ligand (TRAIL) is a member of the TNF family of proteins [3]. Compared with normal cells, tumor cells were initially found to have increased sensitivity to TRAIL, raising hopes that TRAIL would have therapeutic potential as an anti-cancer agent [4, 5]. To date, four distinct cell surface TRAIL receptors have been identified: TRAIL-R1 (DR-4), TRAIL-R2 (DR-5/TRICK2/KILLER), TRAIL-R3 (DcR-1/TRID/LIT), and TRAIL-R4 (DcR-2/TRUNDD). All these receptors have high sequence homology in their extracellular domains. Although TRAIL binds with each of these receptors, the function of their intracellular domains is not uniform. DR-4 and DR-5 have a cytoplasmic region, termed the death domain, which can transmit an apoptotic signal. In contrast, DcR-1 and DcR-2 do not possess a death domain; thus, they cannot form signaling complexes to induce an apoptotic stimulus [6, 7]. In addition, overexpression of either DcR-1 or DcR-2 suppresses TRAIL-induced apoptosis [8, 9].

Head and neck squamous cell carcinomas (HNSCCs) are among the most prevalent tumors worldwide, and approximately 94% of all oral malignancies are squamous cell carcinoma (SCC) [10]. The loss of E-cadherin, a transmembrane protein that provides adhesion between epithelial cells, and the gain of N-cadherin, a transmembrane protein existing in connective tissue, have been recently documented in human SCCs [11, 12]. Loss of E-cadherin protects cancer cells from induced apoptosis [13, 14]. Moreover, it has been reported that N-cadherin might play a role in malignant behaviors, such as promotion of growth and invasion of cancer cells [15–17]. In our previous reports, we validated the association of N-cadherin with the invasive ability in HNSCCs [12]. However, the mechanism underlying the role of N-cadherin in the promotion of cancer cell growth has not yet been clarified, which was the impetus for this study. The findings of the present study indicated that N-cadherin increases cancer cell growth via induction of the anti-apoptosis ability of cancer cells through activation of TRAIL receptors and the caspase pathway.

## RESULTS

### Anti-apoptotic effects of N-cadherin overexpression in KOSCC33A cells

**Figure 1:**
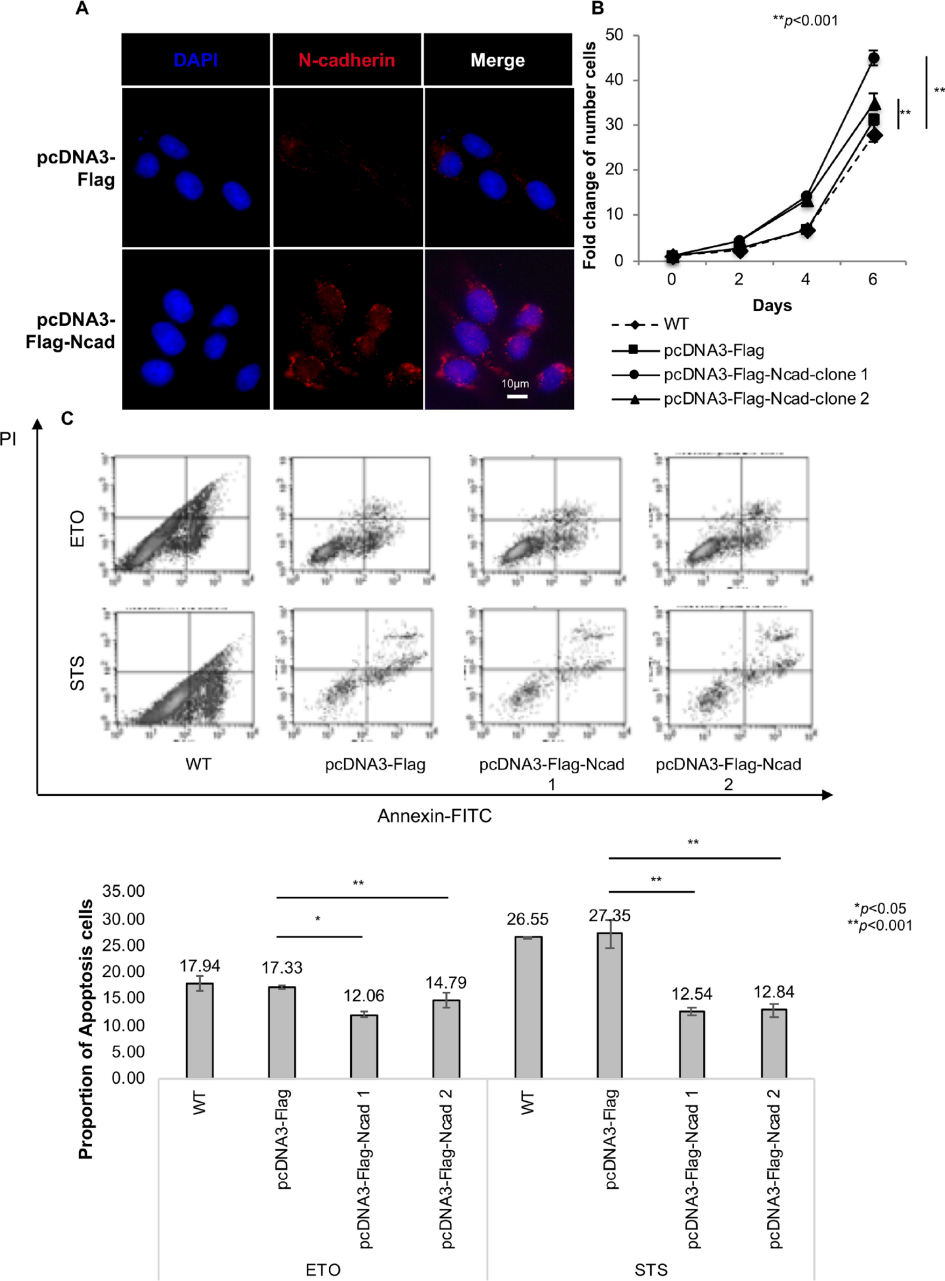
N-cadherin overexpression protects cancer cells from apoptosis (**A**) Transfection of full-length complementary DNA of N-cadherin. N-cadherin expression was examined after transfection into KOSCC33A cells by immunofluorescent staining. (**B**) Growth of KOSCC33 cells with and without N-cadherin overexpression was measured using proliferation assays. Five thousand cells were plated in each well and cultured in DMEM medium. After 2, 4, and 6 days, cells were trypsinized and counted. Similar results were obtained in three independent experiments. Data are presented as the means ± SD (*n* = 3), ^**^p < 0.01, compared with control cells (Student’s *t*-test). (**C**) Flow cytometry to detect apoptosis was performed in triplicate. The results of one representative experiment of three independent experiments are shown. All data are summarized and presented as the mean ± S.D. ^*^p < 0.05, compared with control cells (Student’s *t*-test).

To assess the role of N-cadherin in the regulation of cell proliferation, KOSCC33A cells were transfected with full-length cDNA of *N-cadherin* (Figure 1A), and the number of viable cells was measured at different time points. As reported in Figure 1B, N-cadherin overexpression induced significant growth of KOSCC33A cells, compared to control cells (*p* < 0.001).

Next, the effect of N-cadherin signaling on cancer cell survival was evaluated by comparing apoptosis rates between KOSCC33A cells overexpressing N-cadherin and control cells. Apoptosis rates of KOSCC33A cells with and without N-cadherin overexpression were determined by surface annexin V-FITC using fluorescence-activated cell sorting (FACS). As expected, the mean fluorescence intensity of annexin V-FITC in KOSCC33A cells overexpressing N-cadherin was lower than that of control KOSCC33A cells under STS or ETO treatment (Figure 1C). This finding indicated that N-cadherin overexpression protected KOSCC33A cells from apoptosis, which might explain the increased growth of cancer cells overexpressing N-cadherin.

### N-cadherin overexpression inhibits caspase activation during STS-induced apoptosis

Caspase activation and cleavage of poly (adenosine diphosphate-ribose) polymerase (PARP) play critical roles in the apoptosis process [14, 18, 19]. To determine whether the anti-apoptotic effect of N-cadherin overexpression was associated with the inhibition of caspase activation, cleavage of caspase-3, -6, -7, and -8 and PARP by STS treatment in KOSCC33A cells with and without N-cadherin overexpression was investigated.

**Figure 2:**
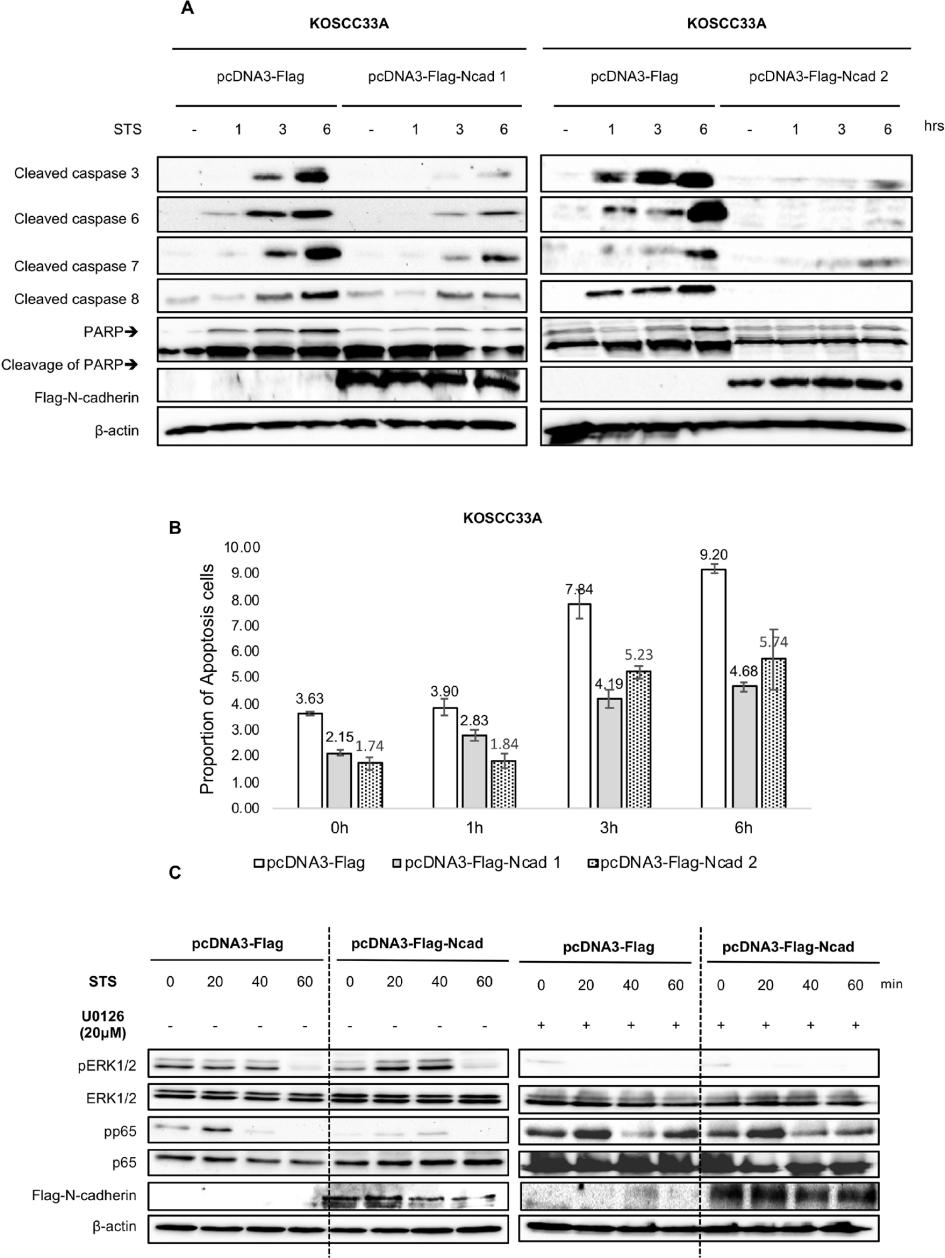
(**A**) N-cadherin overexpression renders KOSCC33A cells resistant to STS-induced apoptosis by inhibiting caspase activation. Expression of caspase-3, -6, -7, -8, PARP cleavage, and ectopic expression of N-cadherin in control KOSCC33A cells and KOSCC33A cells overexpressing N-cadherin was analyzed by western blotting after treatment with 500 nM STS for 1, 3, and 6 h. β-actin expression was used as an internal control. (**B**) Flow cytometry to detect apoptotic cells under 500 nM STS treatment for 1, 3, and 6 h in KOSCC33A control and N-cadherin overexpressing KOSCC33A cells. (**C**) Expression of phospho-ERK, total ERK, phospho-p65, total p65, and ectopic expression of N-cadherin in control KOSCC33A cells and KOSCC33A cells overexpressing N-cadherin were analyzed by western blotting after treatment with 500 nM STS and 20 µM U0126 for 20, 40, and 60 min. β-actin expression was used as an internal control.

Caspase-3, -6, -7 and -8 were activated in KOSCC33A control cells exposed to STS, whereas N-cadherin overexpression in KOSCC33A cells significantly reduced caspase activation. STS-induced caspase-3 activation was almost completely inhibited, whereas activation of caspase-6 and caspase-7 was partially inhibited, and activation of caspase 8 was suppressed a little (Figure 2A). PARP cleavage was also protected by N-cadherin overexpression prior to STS treatment (Figure 2A). Moreover, the percentage of apoptosis cells significantly decreased in N-cadherin overexpressing cells (Figure 2B).

### MAPK/ERK activation and p65/NF-kB suppression in cells overexpressing N-cadherin by STS treatment

To further identify the underlying molecular mechanism linked to chemotherapy resistance, the degree of p38, Akt, and ERK phosphorylation in KOSCC33A cells with and without N-cadherin overexpression was analyzed. As shown in Figure 2B, treatment with 500 nM STS for 20 min resulted in increased phosphorylation of ERK1/2 in KOSCC33A cells overexpressing N-cadherin, as compared to control cells, whereas NF-kB p65 phosphorylation was inhibited (Figure 2C).

To further confirm the inhibitory effect of ERK activation on suppression of nuclear factor (NF)-kB p65 in resistance to STS, the expression levels of phosphorylated p65 and ERK were measured in KOSCC33A cells with and without N-cadherin overexpression by treatment with 500 nM STS and 20 µM U0126, a selective MEK inhibitor. Interestingly, the inhibition of ERK phosphorylation using U0126 induced NF-kB p65 activation in cells overexpressing N-cadherin (Figure 2C). These results indicate that ERK activation negatively regulates NF-kB p65 phosphorylation in a mechanism of STS resistance.

### Correlation between N-cadherin overexpression, DRs, decoy receptors (DcRs), and effects of STS on apoptosis in HNSCC cell lines

**Figure 3:**
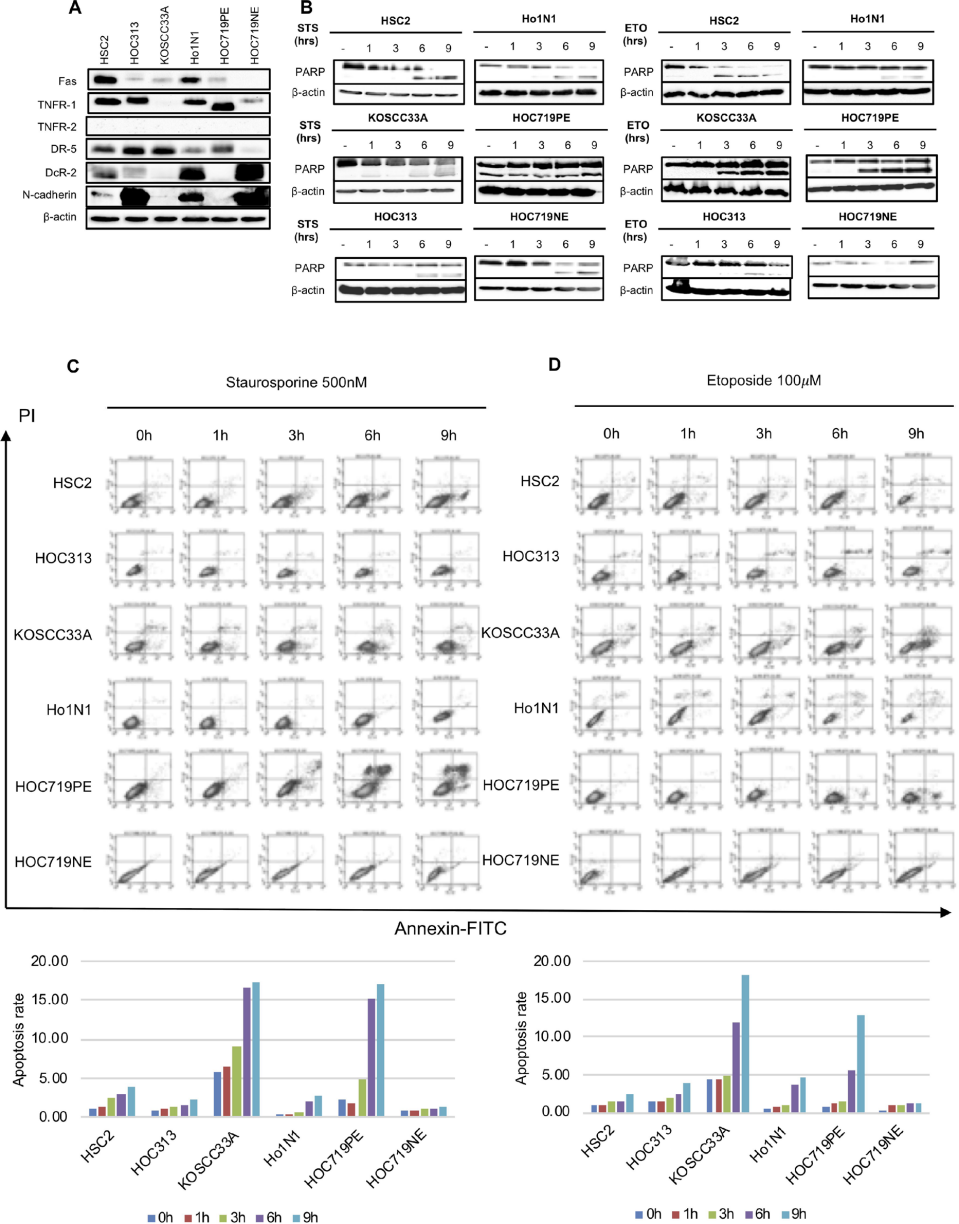
Correlation between expression of N-cadherin and representative DRs in six HNSCC cell lines (HSC2, HOC313, KOSCC33A, Ho1N1, HOC719PE, and HOC719NE) (**A**) The expression of N-cadherin, Fas, TNFR-1, TNFR-2, DR-5, and DcR-2 in HNSCC cell lines was examined by western blotting with β-actin expression as an internal control. (**B**) Cleavage of PARP was examined by western blotting in six HNSCC cell lines after treatment with 500 nM STS and 100 mM ETO for 1, 3, 6, and 9 hrs. β-actin expression was used as an internal control. (**C**) Flow cytometry to detect apoptotic cells in six HNSCC cell lines after treatment with 500 nM STS for 1, 3, 6 and 9 hrs. (**D**) after treatment with 100 mM ETO for 1, 3, 6 and 9 hrs.

The DR pathway is a key apoptotic pathway [20, 21] and N-cadherin is a well-known transmembrane protein [22, 23]. To further clarify the role of N-cadherin in cancer cell growth, we first examined whether there is a correlation between expression of several DRs, such as Fas, TNFR-1, TNFR-2, DR-5, DcR-2, and N-cadherin expression in six cell lines (HSC2, HOC313, KOSCC33A, Ho1N1, HOC719PE, and HOC719NE). Among these cell lines, HSC2, HOC313, Ho1N1, and HOC719NE cells showed positive N-cadherin expression (Figure 3A). Interestingly, all four of these cell lines also expressed DcR-2 (Figure 3A).

Next, we asked whether the existence of N-cadherin in HNSCC inhibits apoptosis. Thus, the effect of STS and ETO on cleavage of PARP in each cell line was examined by checking the time differential of PARP cleavage after STS and ETO treatment for 1, 3, 6, and 9 hrs. Interestingly, in the HNSCC cell lines that do not express N-cadherin (KOSCC33A, and HOC719PE), PARP cleavage occurred after almost 1 hrs of STS treatment or ETO treatment, whereas in the HOC313 and HOC719-NE cells, which strongly express N-cadherin, PARP cleavage was detected after 9 hrs, although expression was very weak (Figure 3B). Moreover, we also found the proportion of apoptosis cells significantly increased in KOSCC33A cells and HOC719PE cells after STS treatment (Figure 3C) or ETO treatment (Figure 3D)

### Correlation between N-cadherin and DcR-2 expression, and TUNEL staining in cancer specimens

**Figure 4:**
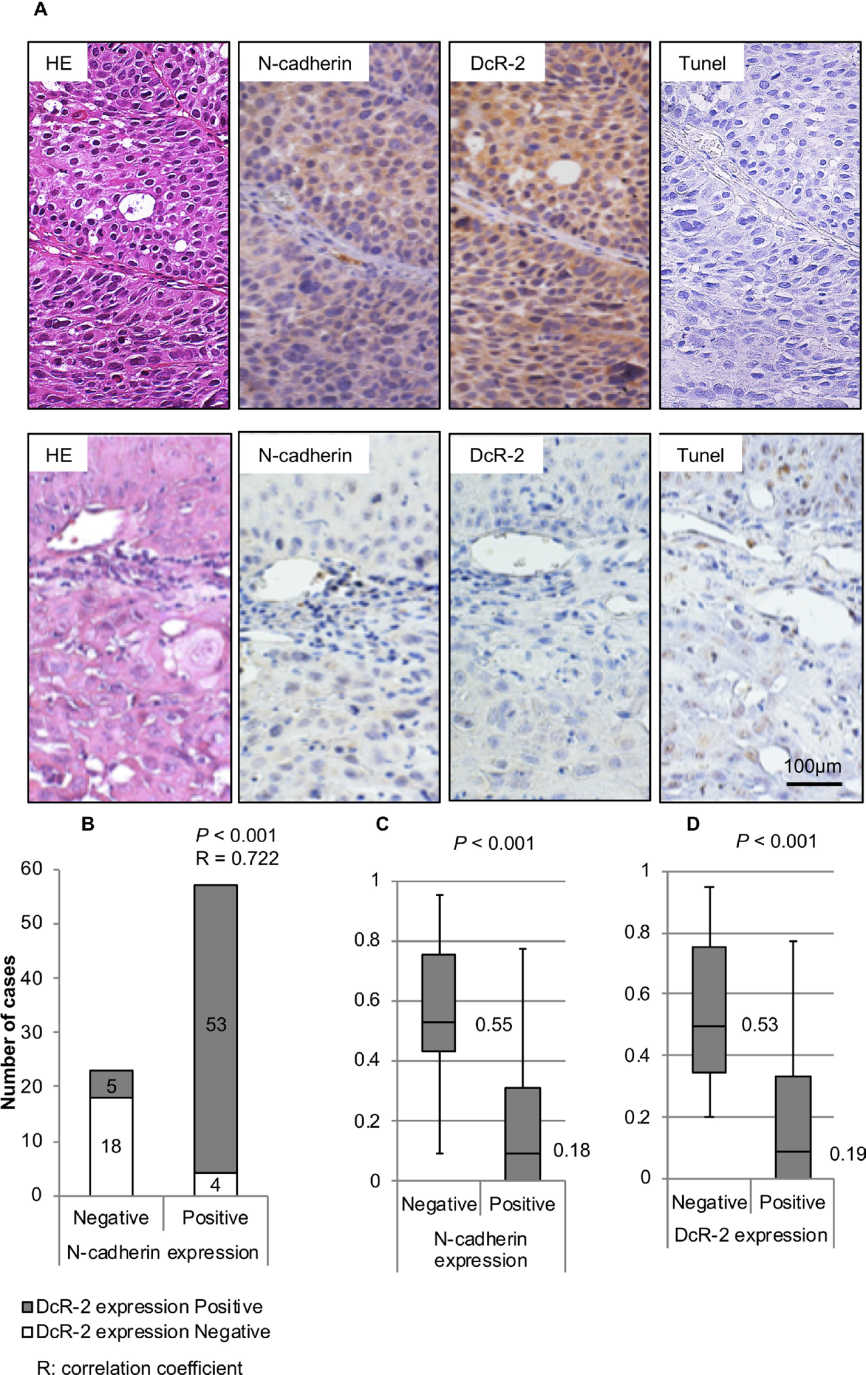
Expression of N-cadherin and DcR-2, and correlations to AI (**A**) The upper panel shows a representative case with HE-staining, positive N-cadherin expression, positive DcR-2 expression, and AI of 0 at a magnification of 200×. The lower panel shows a representative case with HE-staining, negative N-cadherin expression, negative DcR-2 expression, and AI of 0.65 at a magnification of 200×. (**B**) Correlation between N-cadherin and E-cadherin expressions was analyzed using the Spearman test. The graph shows the number of specimens with positive or negative expression of DcR-2 in each group of positive or negative expression of N-cadherin. (**C**) Correlations between N-cadherin expression and AIs were analyzed using the Mann–Whitney *U* test. Box-whisker plots show AI in relation to N-cadherin expression. Median values are shown as heavy lines within the box. (**D**) Correlations between DcR-2 expression and AIs were analyzed using the Mann–Whitney *U* test. Box-whisker plots showed AI in relation to DcR-2 expression. Median values are shown as heavy lines within the box.

A total of 80 HNSCC specimens from 62 men and 18 women were analyzed. The median age at diagnosis was 59 years (range, 22–92 years). Immunohistochemical analysis showed that N-cadherin was expressed in 57 (71.3%) of 80 specimens (Figure 4A), while immunostaining specific for DcR-2 was positive in 58 (72.5%) of 80 specimens (Figure 4A). The AI was 0.29 ± 0.28 (mean ± SD).

Spearman rank correlation analysis showed a statistically significant positive correlation between expression levels of N-cadherin and DcR-2 (*p* < 0.001) (Figure 4B). In regard to AI (evaluated by TUNEL staining), significant correlations were found between decreased AI and positive N-cadherin expression (*t*-test; *p* < 0.001, Mann–Whitney *U* test: *p* < 0.001, Figure 4C) and positive DcR-2 expression (*t*-test; *p* < 0.001, Mann–Whitney *U* test: *p* < 0.001, Figure 4D).

### Enforced expression and knock-down of N-cadherin alters the expression of DR-5 and DcR-2

**Figure 5:**
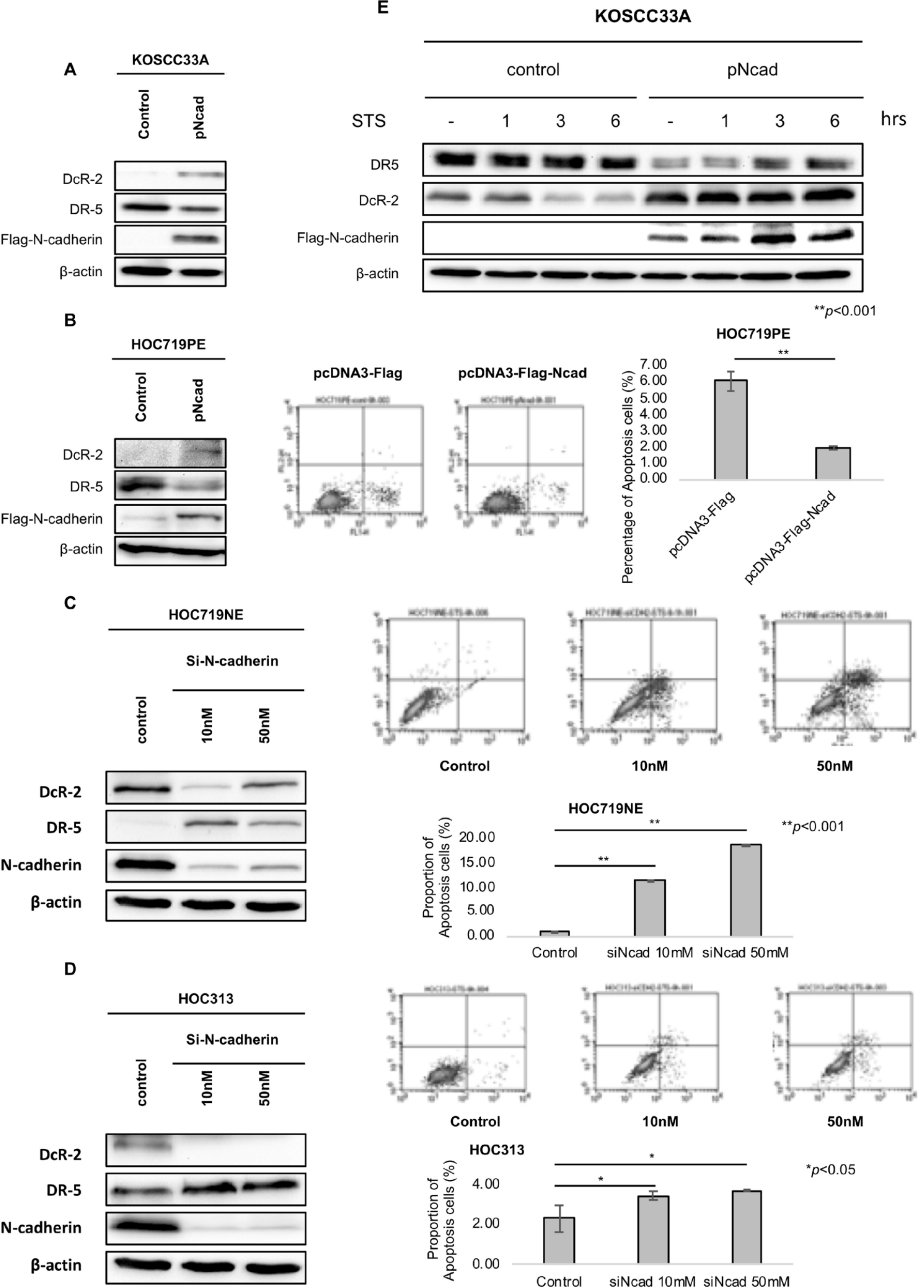
Enforced expression and knock-down of N-cadherin expression alters the expression of DR-5 and DcR-2 (**A**) Expression of DcR-2, DR5, and N-cadherin in control KOSCC33A cells and KOSCC33A cells overexpressing N-cadherin were analyzed by western blotting, with β-actin as an internal control. (**B**) Expression of DcR-2, DR5, and N-cadherin in control HOC719PE cells and transient HOC719PE cells overexpressing N-cadherin were analyzed by western blotting, with β-actin as an internal control. Flow cytometry to detect apoptotic cells in HOC719PE control and N-cadherin overexpressing HOC719PE. (**C**) Expression of DcR-2, DR5, and N-cadherin in control HOC719NE cells and N-cadherin knock-down in HOC719NE cells was analyzed by western blotting, with β-actin used as an internal control. Flow cytometry to detect apoptotic cells in HOC719NE control and N-cadherin knocking down HOC719NE. (**D**) Expression of DcR-2, DR5, and N-cadherin in control HOC313 cells and N-cadherin knock-down in HOC313 cells was analyzed by western blotting, with β-actin used as an internal control. Flow cytometry to detect apoptotic cells in HOC313 control and N-cadherin knocking down HOC313. (**E**) Expression of DR-5 and DcR-2, and ectopic expression of N-cadherin in control KOSCC33A cells and KOSCC33A cells overexpressing N-cadherin were analyzed by western blotting after treatment with 500nM STS for 1, 3, and 6 hrs. β-actin was used as an internal control.

By analyzing the expression levels of N-cadherin, DcR-2, and DR-5 in HNSCC cell lines and specimens, we sought to determine whether N-cadherin is required for DcR-2 expression. Hence, N-cadherin was overexpressed in KOSCC33A cells and HOC719PE and knocked-down in HOC719NE and HOC313 cells. As show in Figure 5A, 5B and Figure 1C, the transfection of N-cadherin resulted in increased expression of DcR-2, decreased expression of DR5, and the decreased proportion of apoptosis cells. Next, N-cadherin was knocked-down using small interfering RNA (siRNA). In HOC719NE and HOC313 cells, N-cadherin siRNA at 10 nM and 50 nM decreased DcR-2 expression, increased DR-5 expression, and the increased percentage of apoptosis cells, as compared to control cells (Figure 5C and 5D), confirming a connection between N-cadherin, DR-5, DcR-2, and apoptosis resistance.

Moreover, KOSCC33A cells with and without N-cadherin expression were treated with STS, and the expression levels of DR-5 and DcR-2 were evaluated by western blot analysis. As expected, DR-5 expression was significantly weaker in cells overexpressing N-cadherin, as compared to control cells, whereas DcR-2 expression was significantly stronger (Figure 5E). These data suggest that N-cadherin controls apoptosis signaling via decreasing DR-5 expression and increasing DcR-2 expression.

### N-cadherin directly interacts with DcR-2

**Figure 6:**
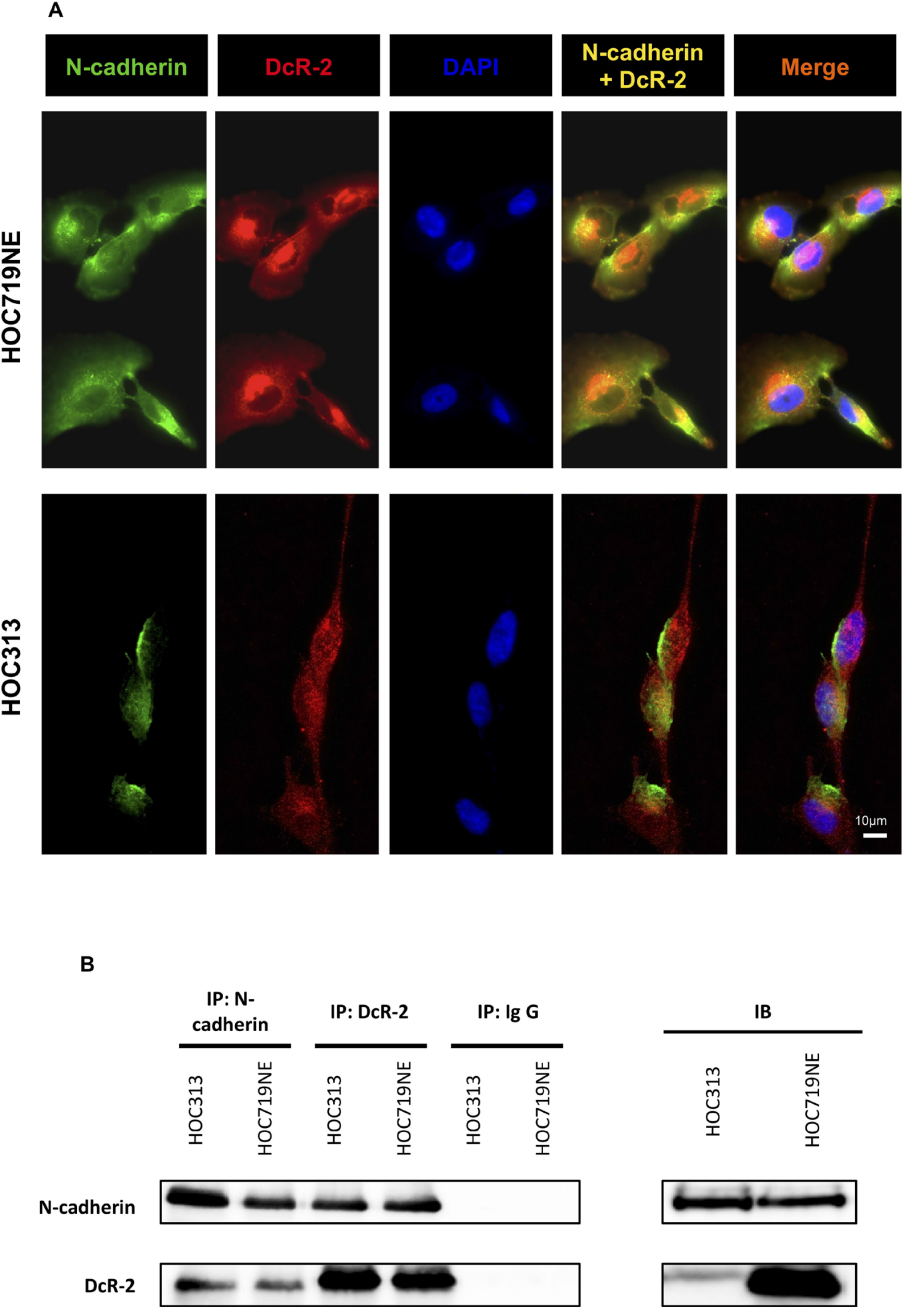
Immunofluorescence microscopy and immunoprecipitation to characterize the physical interactions of N-cadherin and DcR-2 (**A**) Immunofluorescence of HOC313 and HOC719NE cells. The upper panel shows single color channels for expression of N-cadherin in green, DcR-2 in red, DAPI in blue, the overlay of N-cadherin and DcR-2, and the overlay of N-cadherin, DcR-2, and DAPI in HOC719NE cells. The lower panel shows single color channels for expression of N-cadherin in green, DcR-2 in red, DAPI in blue, the overlay of N-cadherin and DcR-2, and the overlay of N-cadherin, DcR-2, and DAPI in HOC313 cells. (**B**) Lysates of HOC313 cells and HOC719NE cells were subjected to immunoprecipitation with N-cadherin antibody (lane 1: HOC313; lane 2: HOC719NE) or with DcR-2 antibody (lane 3: HOC313; lane 4: HOC719NE), and with immunoglobulin G (lane 5: HOC313; lane 6: HOC719NE). Samples were analyzed by immunoblotting as indicated.

Next, possible connections between N-cadherin, DR-5, and DcR-2 were investigated. The expression levels of DcR-2 and N-cadherin in HOC313 and HOC719NE cells, two HNSCC cell lines that strongly express N-cadherin, were investigated by IF staining. Interestingly, expression of DcR-2 was found to overlap (yellow) with that of N-cadherin in HOC313 and HOC719NE cells (Figure 6A), thus indicating possible physical interactions between these proteins. To further clarify these findings, immunoprecipitation of DcR-2 and N-cadherin were performed followed by subsequent staining for N-cadherin and DcR-2. As shown in Figure 6B, interactions between N-cadherin and DcR-2 were detected.

## DISCUSSION

Cadherins are a family of transmembrane proteins that provide anchorage between neighboring cells by interacting with the actin cytoskeleton through catenins [19, 24]. Among them, N-cadherin is usually expressed in neural tissue and fibroblasts, where it is believed to mediate a less stable and more dynamic form of cell-to-cell adhesion [25, 26]. In addition, aberrant N-cadherin expression has recently been implicated in the progression of certain epithelial tumors by promoting invasion and dissemination of cancer cells [12, 27]. The involvement of cadherins in cell survival has been described in both normal and cancer cells. However, the molecular mechanism whereby cadherin adhesion contributes to cell survival remains poorly understood and controversial [13, 28, 29].

The results of our previous studies showed aberrant N-cadherin overexpression and validated the association of N-cadherin with invasive ability and characteristics of HNSCC in clinical samples. These findings prompted us to clone and transfect full-length cDNA of *N-cadherin* into KOSCC33A cells, which showed that N-cadherin overexpression increased cancer cell growth due to induction of anti-apoptosis pathways in KOSCC33A cells.

The relationship between proliferation and cell death might also reflect the fact that cells require survival signals, such as growth factors, cytokines, hormones, or stimuli from adhesion molecules [30]. Past research indicates that there are two main apoptotic pathways: the extrinsic or DR pathway and the intrinsic or mitochondrial pathway [20, 21]. According to our findings, N-cadherin overexpression promoted survival of cancer cells. Therefore, we investigated the ability of these cells to avoid apoptosis through the DR pathway.

Many studies have revealed that various members of the TNF receptor (TNF-R) superfamily and TRAIL receptors represent prototypic entry points for external signals to initiate apoptosis by natural ligands or agonistic antibodies [31]. Within this superfamily, a subset of receptors, known as DRs, are characterized by a cytoplasmic sequence of ∼80 amino acids, termed the death domain, which is essential for apoptosis induction when these receptors are triggered by corresponding ligands [6, 7]. The most extensively studied DRs are Fas (CD95), TNFR-1, TNFR-2, TRAIL-R1 (DR-4), and TRAIL-R2 (DR-5). On the other hand, DR ligands also interact with DcRs that do not possess a death domain and so cannot form signaling complexes. Four DcRs have been identified so far: TRAIL-R3 (DcR-1), TRAIL-R4 (DcR-2), DcR-3, and osteoprotegerin [8, 9]. Therefore, we examined the expression of representatively known DRs and DcRs, such as Fas, TNF-R1, TNF-R2, DR-5, and DcR-2, and the correlation between these receptors with N-cadherin expression in HNSCC cell lines.

Fas (CD95), a member of TNF family, was found in normal and malignant breast epithelial cell lines (Keane *et al.*, 1996), and in samples of oral SCC, yet Fas expression was not significantly associated with tumor size or differentiation grade [32]. In the present study, almost all examined HNSCC cell lines showed Fas expression and, interestingly, HNSCC cell lines, which are positive for Fas expression, are also positive for DR-5 expression.

Most TNFR superfamily members function as transmembrane signal transducers that are activated upon binding of a ligand. However, some receptors act as decoys that compete for the ligands [7, 33]. Among these DcRs, DcR-2 contains an extracellular TRAIL-binding domain, a transmembrane domain, and a truncated cytoplasmic death domain [34]. Therefore, this receptor does not induce apoptosis, but rather has been shown to play an inhibitory role in TRAIL-induced cell apoptosis. In addition, our previous studies showed that N-cadherin was expressed in the membrane and cytoplasm of cancer cells, which is why we investigated whether N-cadherin augments the function of DRs and/or DcRs. We found that HNSCC cell lines, which express N-cadherin, also express DcR-2, whereas the expression of DcR-2 and DR-5 is reversed in HNSCC cell lines.

In the current study, a significant positive relationship was noted between expression levels of N-cadherin and DcR-2 in HNSCC specimens. Besides, the results of our study provide evidence that specimens with a lower AI showed greater expression of N-cadherin and/or DcR-2. In many studies, the AI was found to be a significant prognostic factor for longer survival [35]. Furthermore, the enforced DcR-2 expression reduced cell sensitivity to chemotherapeutic agents and thus affected chemosensitivity [34].

In this study, we used STS, a well-known inducer of apoptosis in a wide range of cell lines [36]. The present results show that in KOSCC33A cells with and without N-cadherin expression, STS induced a time-dependent difference in cleavage of involved caspases and PARP, which are expressed as inactive proenzymes and becomes activated by proteolytic processing when cells receive an apoptosis-inducing signal [37, 38]. Therefore, N-cadherin overexpression in KOSCC33A cells reduces sensitivity of the cells to STS. Moreover, an opposite correlation between DcR-2 and DR-5 was clearly shown by the STS-resistance status of KOSCC33A cells overexpressing N-cadherin.

Consistent with these findings, we considered the possibility of a connection between N-cadherin, DR-5, and DcR-2. Thus, we explored the direct interaction between N-cadherin and DcR-2 by IF staining and immunoprecipitation. Based on the strong evidence of our findings, we presumed that N-cadherin is directly associated with DcR-2 to provide anchorage for DcR-2 in the cell membrane to compete with DR-5 to inhibit apoptosis.

To date, various mechanisms that interfere at different levels of apoptosis signaling have been studied. In a wide variety of cell types, increased resistance to apoptosis can be induced by a range of different alterations, such as the activation/up-regulation of ERK1/2 and Akt mitogenic signaling pathways. Other studies have shown that growth factors and cytokines activate Akt [39] and inhibit pro-apoptotic Bcl-2, thereby interfering with the apoptotic machinery [30]. However, our results showed that N-cadherin overexpression mediated anti-apoptosis through the ERK/MAP kinase pathway, but not the Akt pathway. MAP kinases are composed of ERK, JNK, and p38. Activation of the JNK and p38 signaling pathways tends to promote apoptosis, whereas activation of the ERK signaling pathway tends to inhibit apoptosis [40, 41].

Moreover, the results of the current study showed that NF-kB p65 was a target of the MAP kinase pathway in the regulation of apoptosis. NF-kB was first identified as a B-cell nuclear factor that regulates apoptosis [42, 43]. Furthermore, NF-kB is also actively involved in signaling inflammation, cell transformation, and anti-apoptosis processes. The exact signaling function of the p65/ERK complex is currently unknown. P65 is a well-defined subunit of NF-kB that forms DNA binding homodimers or heterodimers and can be activated by oxidative damage or crosstalk with 53BP2, a protein identified by interactions with wild-type p53 and Bcl2 to inhibit cell death [41]. In this study, the MEK/ERK pathway was shown to act upstream of NF-kB.

**Figure 7:**
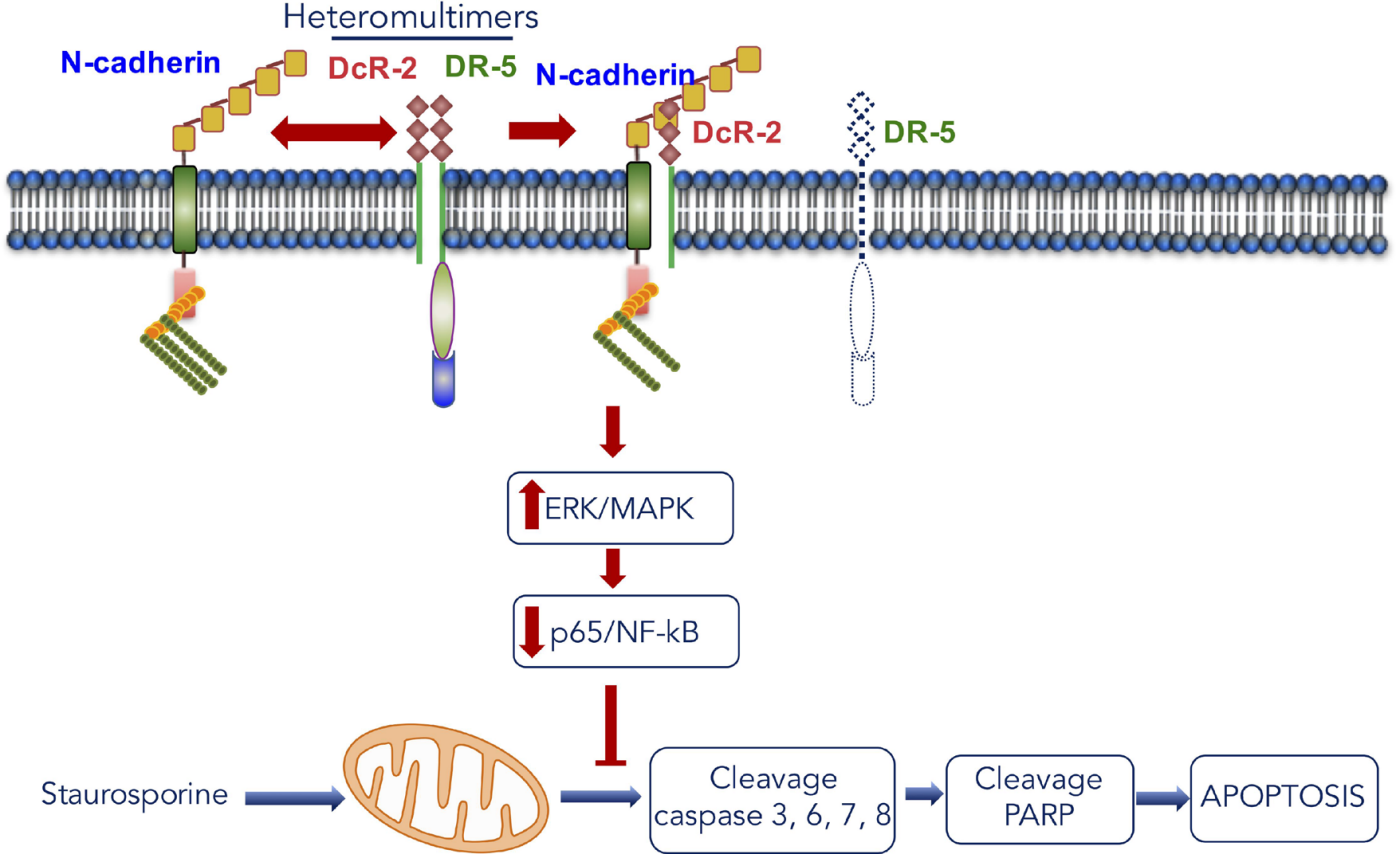
Scheme for the mechanism of apoptosis induction caused by the interaction between N-cadherin and DcR-2 in head and neck cancer

In summary, our findings showed that N-cadherin is an important moderator of anti-apoptosis signaling through the interaction with DcR-2 and plays a potentially significant role in cross-regulation between cell-cell adhesion and DR function (Figure 7). Moreover, these results revealed a connection between N-cadherin, DR-5, Dc-R2, MAPK/ERK, and NF-kB/p65, which will provide essential information on the mechanism underlying tumor resistance to chemotherapy (Figure 7). In addition, these results indicate that a reduction in p65/ERK complexes is linked to anti-apoptosis due to N-cadherin overexpression in HNSCC cells (Figure 7) and molecular targeting of MAPK/ERK activation to suppress NF-kB activity holds promise as an effective approach to re-sensitize resistant tumor cells.

## MATERIALS AND METHODS

### Patient specimens

Eighty paraffin-embedded HNSCC specimens were obtained from Hiroshima University Hospital (Hiroshima, Japan) following approval by the Ethics Committee of Hiroshima University. The clinical details were gathered from the surgical records of patients. Only specimens from patients who did not receive radio-chemotherapy were selected to avoid possible influences of treatment. The experiments were undertaken with the understanding and written consent of each subject.

### Cell lines and cell culture

Six HNSCC cell lines were used for this study. HSC2 and Ho1N1 cells were provided by the Japanese Collection of Research Bioresources Cell Bank (Osaka, Japan). HOC313, HOC719PE, and HOC719NE cells were provided by Prof. Kamata (Hiroshima University, Japan) [44]. KOSCC33A cells were obtained from Dr. S.P. Hong (Seoul National University, Seoul, South Korea) [45]. All cells were maintained in Roswell Park Memorial Institute 1640 medium (RPMI 1640) or Dulbecco’s modified Eagle’s medium (DMEM) (Nissui Pharmaceutical Co., Tokyo, Japan) supplemented with 10% heat-inactivated fetal bovine serum (Invitrogen Corporation, Carlsbad, CA, USA), 100 U/mL penicillin–streptomycin (Gibco, Carlsbad, CA, USA) under an atmosphere of 5% CO_2_ at 37° C.

### Antibodies and reagents

Staurosporine (STS), an apoptosis inducer, and U0126, an inhibitor of MEK, were purchased from Enzo Life Sciences, Inc. (Farmingdale, NY, USA). Etoposide (ETO), an apoptosis inducer, was purchased from Tokyo Chemical Industry Co., LTD. (Tokyo, Japan).

The following antibodies were used for western blot, immunoprecipitation, and immunofluorescence (IF) analysis: anti-N-cadherin (BD Transduction Laboratories, Franklin Lakes, NJ, USA), anti-Flag (Sigma Aldrich, MO, USA), anti-DR-5, anti-DcR-2, anti-Fas, anti-TNFR-1, and anti-TNFR-2 (Cell Signaling Technology, Inc., Danvers, MA, USA), anti-phospho extracellular signal-regulated kinase (ERK)1/2, anti-ERK1/2 (Santa Cruz Biotechnology, Inc., Santa Cruz, CA, USA), anti-phospho p38, anti-p38, anti-phospho Akt, anti-Akt, anti-phospho NF-kB p65, anti-NF-kB p65, anti-cleavage PARP (Cell Signaling Technology, Inc.), and anti-β actin (Sigma-Aldrich Corporation, St. Louis, MO, USA).

HOC313 and HOC719NE cells were transfected with N-cadherin small interfering RNA (si-CDH2) (Ambion, Life Technologies, Foster City, CA, USA) using Oligofectamine reagent (Invitrogen Corporation) following the manufacturer’s instructions.

### Immunohistochemical (IHC) staining and evaluation

The IHC staining procedure has been described [27]. IHC staining was performed on 4.5-µm sections. Primary antibodies against N-cadherin and DcR-2 were diluted in PBS to 1:100, stored at 4° C overnight in a humidified container, and then labelled with diaminobenzidine (DAKO, Japan). Immunostaining was evaluated based on the proportion of stained cancer cells and the location of the staining. The samples were then divided into two groups: positive or negative staining (>50% or <50% of stained cancer cells, respectively).

### Terminal deoxynucleotidyl transferase dUTP nick end labeling (TUNEL) analysis and quantification of the apoptotic index (AI)

Apoptotic cells were identified by the TUNEL assay. Dewaxed and rehydrated specimens were incubated in 10 µg/mL proteinase K (Qiagen, Venlo, Netherlands) for 15 min at 37° C and then treated with 3% H_2_O_2_ in methanol for 30 min at room temperature. After adding equilibration buffer for 5 min at room temperature, terminal TdT enzyme was pipetted on to the sections, which were then further incubated at 37° C in humidified chamber for 60 min. The reaction was stopped by incubating the sections in stop buffer for 30 min at 37° C. Horseradish peroxidase-conjugated anti-fluorescein isothiocyanate (FITC) was added to the slides, which were then incubated for 30 min at 37° C, stained with diaminobenzene (DAKO, Japan) for 10 min, and counterstained with hematoxylin. The AI was determined by calculating the percentage of positive cells in at least 500 cells at 400× magnification.

### Plasmid construction and transfection

The full-length *N-cadherin* sequence was PCR-amplified from fibroblast genomic DNA using the primers CDH2–F 5′-CCG CTC GAG CGG ATG TGC CGG ATA GCG GGA GCG-3′ and CDH2–R 5′-CGC GGA TCC GCG GTC ATC ACC TCC ACC ATA CAT-3′. cDNA was subcloned into pcDNA3-Flag, which was a gift from Stephen Smale [46], using the restriction endonucleases *Xho*I and *Bam*HI, and then transformed into recombination-competent *Escherichia coli* cells. The vector pcDNA3-Flag-N-cad was transfected into cells using Fugene 6 transfection reagent (Roche Diagnostics Corporation, IN, USA) following the manufacturer’s instructions, and stabilized by G418 (Thermo Fisher Scientific, MA, USA).

### Immuno-fluorescent (IF) staining

Cells grown on coverslips were subjected to IF staining and then fixed with 4% paraformaldehyde. Primary antibodies against N-cadherin and DcR-2 were diluted in 10% DMEM (1:100) for 1 h and then labelled with the appropriate secondary antibody. DNA was visualized by staining with 4′,6-diamidino-2-phenylindole (DAPI). Immunostained cells were recorded using a Zeiss Axioplan 2 epifluorescent/light microscope (Carl Zeiss Microscopy, LLC, Thorwood, NY, USA) equipped with a charge coupled device camera.

### Proliferation assay

Cells were plated in a 24-well-plate at a density of 5,000 cells per well and cultured in DMEM. After 2, 4 and 6 days, the cells were trypsinized and counted using a cell counter (Coulter Electronics, Ltd., Buckinghamshire, England). Each experiment was repeated at least three times in triplicate wells. Growth curves showing means and standard deviations were constructed.

### Apoptosis assay

Adherent and supernatant cells were washed two times with PBS to remove any remaining media. Externalization of phosphatidylserine to the outer layer of the cell membrane was examined using an Annexin V-FITC apoptosis detection kit (Medical & Biological Laboratories Co., LTD, Tokyo, Japan).

Cells were washed, suspended in the Annexin V binding buffer, and stained with FITC-conjugated annexin V antibody and Propodium Iodide for 15 min at room temperature. Samples were immediately analyzed with a FACScan flow cytometer with Cell Quest software (BD GmbH, Heidelberg, Germany). This method allows differentiation of early (Annexin V-FITC-positive/propidium iodide-positive) apoptosis cells. The apoptosis rate was defined as the percentage of the Annexin V-FITC-positive cells. All assays were performed in triplicate.

### Western blot and immunoprecipitation analysis

The cells were lysed in a buffer containing 50 mM Tris (pH 7.5), 250 mM NaCl, 0.1% Triton X (Sigma-Aldrich Corporation), 1 mM ethylenediaminetetraacetic acid, 50 mM NaF, and protease and phosphatase inhibitor cocktails (Thermo Scientific™ Haltliquid), and centrifuged at 12,000 *g* for 20 min. Supernatants were immunoprecipitated using the Dynabeads Protein G immunoprecipitation kit (Invitrogen Corporation), according to the manufacturer’s instructions, with N-cadherin antibody (610920, BD Transduction Laboratories), and DcR-2 antibody (D13H4, Cell Signaling Technology, Inc.). Beads were collected by brief centrifugation, washed four times with buffer, boiled in sodium dodecyl sulfate (SDS) gel loading buffer, separated by 10% SDS-polyacrylamide gel electrophoresis, and transferred onto a nitrocellulose membrane (Schleicher & Schuell BioScience GmbH, Dasse, Germany) by electroblotting. The membrane was blocked with PBS buffer (137 mM NaCl, 8.1 mM Na_2_HPO_4_•12 H_2_O, 2.68 mM KCl, 1.47 mM KH_2_PO4) containing non-fat dry milk powder. The primary antibodies were diluted in PBS containing 5% non-fat dry milk powder and incubated overnight at 4° C. Then, the strips were washed and incubated with the secondary antibody for 30 min at room temperature. The immuno-complex was visualized with an enhanced chemiluminescence HRP substrate for western blot analysis using the Western Lightning Plus ECL kit (PerkinElmer, Inc., Waltham, MA, USA) using a Molecular Imager^®^ system (BioRad Laboratories, Hercules, CA, USA).

### Statistical analysis

The SPSS 21.0 statistical software package (SPSS, Inc., Chicago, IL, USA) was used to perform all statistical analyses. The Spearman rank correlation coefficient was used to identify correlations between immunostaining parameters. Statistical analysis of the AI and expression of N-cadherin and DcR-2 was performed using the Mann–Whitney *U* test. A probability (*p*) value < 0.05 was considered statistically significant.

## Abbreviations

TNF: tumor necrosis factor
DRs: death receptors
TRAIL: necrosis factor-related apoptosis-inducing ligand
DR-4: death receptor 4
DR-5: death receptor 5
DcR-1: decoy receptor 1
DcR-2: decoy receptor 2
HNSCCs: head and neck squamous cell carcinoma
STS: staurosporine
ETO: etoposide
